# 5-Azacitidine induces demethylation of *PTPL1* and inhibits growth in non-Hodgkin lymphoma

**DOI:** 10.3892/ijmm.2015.2269

**Published:** 2015-07-01

**Authors:** WENMING WANG, JING WANG, MIN LI, JIANMING YING, HONGMEI JING

**Affiliations:** 1 Department of Hematology, Peking University Third Hospital, Beijing 100191, P.R. China; 2 Department of Pathology, Peking University Third Hospital, Beijing 100191, P.R. China; 3 Department of Pathology, Cancer Institute and Hospital, Chinese Academy of Medical Sciences, Beijing 100021, P.R. China

**Keywords:** *PTPL1*, mehthylation, lymphoma, diffuse large B-cell lymphoma, 5-azacytidine

## Abstract

Non-Hodgkin lymphoma (NHL) consists of various lymphoid malignancies with a diverse clinical pathology and biological characteristics. Methylation of cytosine residues by DNA methyltransferases at CpG dinucleotides in the promoter region of the genes is a major epigenetic modification in mammalian genomes that can have profound effects on gene expression. The *PTPL1* methylation pattern was screened by methylation-specific polymerase chain reaction (MSP) in 7 lymphoma-derived cell lines and in 47 samples of diffuse large B cell lymphoma (DLBCL). The *PTPL1* gene was hypermethylated in the CA46, Raji, Jurkat and DB cell lines; however, it was unmethylated in the Hut78, Maver and Z138 cell lines. The expression of *PTPL1* mRNA was re-inducible by 5-azacytidine (5-Aza), an agent of DNA demethylation. The methylations were detected in 59.6% of DLBCL versus 6.3% in reactive lymph node proliferation. Therefore, the present data showed that *PTPL1* was epigenetically regulated in NHL suggesting an involvement of the *PTPL1* tumor-suppressor genes in NHL, and highlights 5-Aza as a potential therapeutic candidate for NHL.

## Introduction

Non-Hodgkin lymphoma (NHL) is a common hematological cancer with multiple subtypes, derived from various differentiation stages of the B cell lineage. Burkitt lymphoma (BL) is the most common NHL subtype (69%), followed by lymphoblastic lymphoma, diffuse large B cell lymphoma (DLBCL) and anaplastic large-cell lymphoma, accounting for 18.3, 10.6 and 2.1% of the cases, respectively (1). Although high-dose multiagent chemotherapy and targeted agents induce high remission rates in patients with previously untreated NHL, relapse and drug resistance within a few years is common. Therefore, discovering new therapeutic agents for NHL is required (2).

Cancer cells develop acquiring a set of functional capabilities for malignant growth, such as self-sufficiency in growth signals, insensitivity to growth-inhibitory signals and evasion from apoptosis (3,4). These essential alterations in cell physiology are achieved by the constitutive activation of oncogenes and the loss of function of the tumor-suppressor genes (5). Genetic and epigenetic mechanisms can all contribute to the inactivation of tumor-suppressor genes (6). Methylation of cytosine residues by DNA methyltransferases (DNMTs) at CpG dinucleotides in the promoter region of genes is a major epigenetic modification in mammalian genomes that can have profound effects on gene expression (7,8). One study has shown that DNMTs, including DNMT1, DNMT3A and DNMT3B are overexpressed in 48, 13 and 45% of *de novo* DLBCLs, respectively, which correlates with advanced clinical stage (9). In addition, the therapeutic efficacy of the demethylating agents, such as decitabine and 5-azacytidine (5-Aza), can induce significant clinical responses and even prolong the survival of patients with higher-risk myelodysplastic syndrome (10).

*PTPL1* maps to the human chromosomal locus 4q21, and encodes a cytoplasmic tyrosine phosphatase with a molecular mass of 270 kDa with roles in numerous physiological and pathological processes. Among the potential roles in carcinogenesis, the *PTPL1* gene product can impact cancer development through its capacity to counteract the activity of oncogenic tyrosine kinases or its inhibitory interaction with the death receptor Fas (11). Several studies have shown that hypermethylation of the *PTPL1* gene promoter is involved in various types of cancers, such as non-small cell lung cancer (11), esophageal cancer, gastric and hepatocellular tumors (2,12).

The aim of the present study was to analyze *PTPL1* methylation patterns in a broad spectrum of NHL-derived cell lines and *de novo* DLBCL samples. Epigenetic regulation of *PTPL1* was confirmed in experiments with a DNA demethylating agent. The results obtained from the study significantly contribute towards an improved understanding of the role of *PTPL1* as a tumor-suppressor gene in NHL, and 5-Aza may offer a potential new therapeutic approach to improve the poor outcomes associated with NHL.

## Materials and methods

### Human cell lines

#### Cell culture

The study included 7 cell lines, Hut78 (cutaneous T cell lymphoma cell line), Maver, Z138 (mantle cell lymphoma cell lines), CA46, Raji (Burkitt lymphoma cell lines), Jurkat (acute T cell lymphoma cell line) and DB (DLBCL cell line). All the cell lines, except CA46, were maintained in RPMI-1640 supplemented with 10% fetal bovine serum (FBS) (HyClone, Logan, UT, USA) and 1% antibiotics (Gibco-Invitrogen, Carlsbad, CA, USA). CA46 was maintained with RPMI-1640 supplemented with 20% FBS (HyClone) and 1% antibiotics (Gibco-Invitrogen). Cells were incubated at 37°C in a humid atmosphere at 5% CO_2_ and split every 2–3 days depending on cell density.

#### In vitro cytotoxicity assays

Raji and Jurkat cells in the logarithmic growth phase were inoculated in a 96-well plate, with 100 *µ*l/well and a cell suspension density of 2.5×10^5^/ml. The cells were randomly divided into the control and test groups medially with 4 duplicates/group. They were subsequently treated with 5-Aza at 0.1, 0.5, 1, 2, 5, 10, 20 and 50 *µ*mol/l for 24, 48 and 72 h, respectively. CCK-8 (10 *µ*l; Dojindo, Kumamoto, Japan) accompanied every sampling in each well. After 2 h of incubation, the absorption value (A) of each well was detected at the wavelength of 450 nm in a Quant spectrophotometer. Drug-free wells were used as a control and the no-cell wells with the same amounts of 5-Aza were used as blank controls. Cell inhibition rate (I%) was calculated using the following equation: I% = [A(control) − A(treated)/A(control) − A(blank)] × 100%

#### Treatment with 5-Aza

5-Aza dissolved in normal saline was used to verify the effect on *PTPL1* expression. Three cells lines (CA46, Raji and Jurkat) were seeded at a density of 2.5×10^5^ cells/ml and 5-Aza was added at a final concentration of 20 *µ*mol/l for CA46, 15 *µ*mol/l for Raji and 3.5 *µ*mol/l for Jurkat. Cells were randomly assigned into 3 groups: Negative control group (added in normal saline), 5-Aza-24 h group (treated with 5-Aza for 24 h) and 5-Aza-48 h group (treated with 5-Aza-Cdr for 48 h). Cells were harvested, respectively, to prepare DNA and RNA.

#### DNA extraction and bisulfite conversion

Genomic DNA was extracted by the E.Z.N.A^®^ Tissue DNA kit (Omega Bio-Tek, Lilburn, GA, USA). DNA (200 ng) in a volume of 1–5 *µ*l and was subjected to treatment with sodium bisulfite using a CpGenome DNA modification kit (Epigentek, Farmingdale, NY, USA), according to the manufacturer's instructions. Modified DNA was stored at −80°C until use.

#### Methylation-specific polymerase chain reaction (MSP)

Modified DNA was subjected to two separate PCRs. MSP primers were designed to amplify the methylated or unmethylated alleles, and the Methylamp Universal Methylated DNA kit (Epigentek) was used as a positive control. Promoter meth-ylation status was analyzed by MSP using methylated and unmethylated gene-specific primers for *PTPL1* (12). Primers for *PTPL1* were 5′-CGAGTAGTTTTA GCGGTTAC-3′ (sense) and 5′-AAAACCTTCTAACGCGAA CGA-3′ (antisense) for the methylated reaction and 5′-TGTGAGTAGTTTTAGTGGTTAT-3′ (sense) and 5′-CAAAACCTT CTAACACAAACAA-3′ (antisense) for the unmethylated reaction. These primer sets were designed to amplify 160 and 163 bp, respectively. The methylation promoter was: 95°C for 5 min; 40 cycles of 95°C for 50 sec, 58°C for 1 min and 72°C for 1 min; and a final extension at 72°C for 10 min; the unmethylation promoter was: 95°C for 5 min; 40 cycles of 95°C for 50 sec, 60°C for 1 min and 72°C for 1 min; and a final extension at 72°C for 10 min. Amplified products were visualized under an ultraviolet gel imaging system using the GeneSnap System (Multi Genius; Syngene, Cambridge, UK) following electrophoresis in 2% agarose gels containing the GelRed Nucleic Acid Gel Stain. For each case, MSP results were scored when a clearly visible band on the electrophoresis gel with the methylated and/or the unmethylatated primers were observed. Results from triplicate experiments were used to determine methylation status.

#### RNA isolation and reverse transcription-polymerase chain reaction (PCR)

RNA was isolated using TRIzol (Gibco-Invitrogen), according to the manufacturer's instructions. Total cellular RNA (1 *µ*g) was reverse transcribed using the GoScript™ Reverse Transcription system (Promega, Madison, WI, USA). Primers were: *PTPL1* forward, 5′-GCG CTCCAGTAGCAGGAC-3′ and reverse, 5′-TCATCTGTA AATGACACACTAC-3′; and glyceraldehyde 3-phosphate dehydrogenase (*GAPDH*; as a control) forward, 5′-GGAGCG AGATCCCTCCAAAAT-3′ and reverse, 5′-GGCTGTTGT CATACTTCTCATGG-3′. Amplified products were visualized under an ultraviolet gel imaging system using the GeneSnap System (Multi Genius; Syngene) following electrophoresis in 2% agarose gels containing a GelRed Nucleic Acid Gel Stain.

#### Western blot analysis

Protein was extracted from Hut78, Maver, Z138, CA46, Raji, Jurkat and DB cell lines. Protein concentrations of cells were determined using a bicinchoninic acid protein assay kit (Applygen Technologies Inc., Beijing, China). Western blot analyses were performed using the following primary antibodies: Anti-PTPL1 (1:200; sc-15356) and anti-β-actin (1:1,000; sc-130656) (both from Santa Cruz Biotechnology, Inc., Dallas, TX, USA). Lysates (60 *µ*g) were resolved on sodium dodecyl sulfate-polyacrylamide gel electrophoresis gels (PTPL1 8% and β-actin 10%) and transferred to NC membranes. Membranes were blocked with 5% bovine serum albumin in Tris-buffered saline and Tween 20 and primary antibodies (Abs) were added overnight. Fluorescently labeled secondary antibodies (1:10,000) were used and the membranes were scanned using the Odyssey Infrared Imaging system (both from LI-COR Biosciences, Lincoln, NE, USA).

### Patients

#### Patient selection

The formalin-fixed paraffin-embedded (FFPE) tissues of 47 DLBCL patients and 16 reactive lymph nodes (as control) were evaluated. The archived FFPE tissues were obtained from the Department of Pathology, Peking University Third Hospital (Beijing, China). The patients were diagnosed according to the criteria of the 2008 World Health Organization classification and were clinically staged according to the Ann Arbor classification. Clinical outcomes were evaluated according to the standard international criteria.

#### DNA extraction, bisulfite conversion and MSP

Genomic DNA of 47 patient samples and 16 reactive lymph nodes were extracted using the E.Z.N.A^®^ FFPE DNA kit (Omega Bio-Tek). DNA (200 ng) in a volume of 1–5 *µ*l was subjected to treatment with sodium bisulfite using a CpGenome DNA modification kit (Epigentek). The reaction system and reaction conditions of MSP were the same as the experimental cell lines.

#### Statistical analysis

Statistical analyses were carried out with Social Sciences software (SPSS, version 20.0; IBM Corp., Armonk, NY, USA). Pairwise correlations between the methylation status of DLBCL and control patients, and the germinal center phenotype (GCB) and non-GCB patients were investigated by χ ^2^ test or Fisher's exact test where appropriate. Statistical significance was set at the two-sided 5% comparison wise. P<0.05 was considered to indicate a statistically significant difference.

## Results

### Human cell lines

#### Analysis of PTPL1 gene methylation in lymphoma cell lines

**Figure 1:**
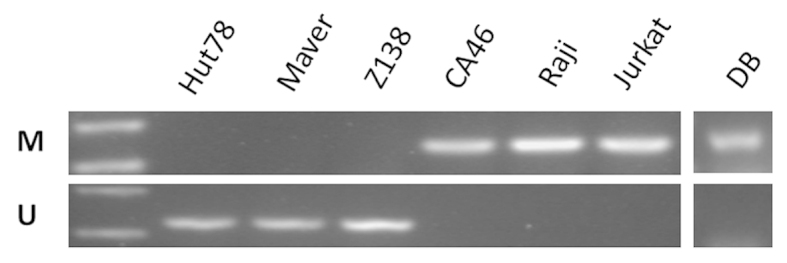
Representative analyses of the methylation of *PTPL1* in multiple lymphoma cell lines. M, methylated; U, unmethylated; Hut78, cutaneous T cell lymphoma; Maver and Z138, mantle lymphoma; CA46 and Raji, Burkitt's lymphoma; Jurkat, acute T cell lymphoma cell line; DB, diffuse large B cell lymphoma.

The *PTPL1* methylation pattern was analyzed by MSP. Following bisulfite conversion of DNA, the methylation status of *PTPL1* was determined with MSP in the lymphoma cell lines. According to MSP, *PTPL1* was methylated in the CA46, Raji, Jurkat and DB cell lines and unmethylated in the Hut78, Maver and Z138 cell lines (Fig. 1).

#### PTPL1 mRNA expression in lymphoma cell lines

**Figure 2:**
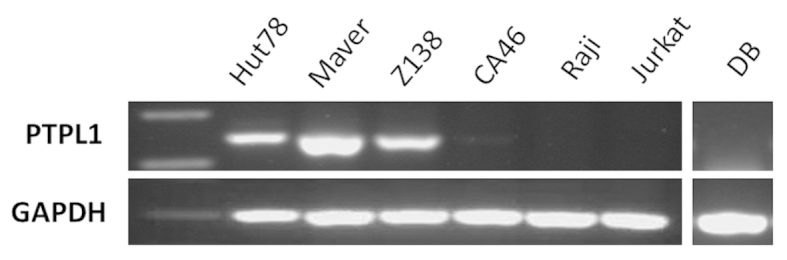
Expression of *PTPL1* mRNA in lymphoma cell lines. *PTPL1* mRNA expression was detected by semi-quantitative reverse transcription-polymerase chain reaction, with *GAPDH* as a control. The expression of *PTPL1* mRNA was ubiquitously expressed at different levels in Hut78, Maver and Z138 cells, but silenced in CA46, Raji, Jurkat and DB cells.

To evaluate the correlation between methylation of the *PTPL1* and *PTPL1* transcription, reverse transcription PCR was performed with cDNA from the lymphoma cell lines. The expression of *PTPL1* mRNA was ubiquitously expressed at different levels in Hut78, Maver and Z138 cells, but silenced in CA46, Raji, Jurkat and DB cells (Fig. 2).

#### PTPL1 protein expression in lymphoma cell lines

**Figure 3:**
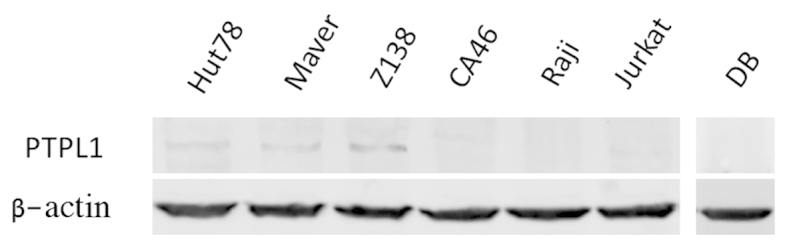
Western blott analysis of the PTPL1 protein in Hut78, Maver, Z138, CA46, Raji, Jurkat and DB cell lines, with β-actin as a control. The PTPL1 protein was ubiquitously expressed at different levels in Hut78, Maver and Z138 cells, but silenced in CA46, Raji, Jurkat and DB cells.

Further examination was performed on the PTPL1 protein. The expression of the PTPL1 protein was ubiquitously expressed at different levels in Hut78, Maver and Z138 cells, but silenced in CA46, Raji, Jurkat and DB cells (Fig. 3). In the majority of the lymphoma cell lines, *PTPL1* gene expression was inversely correlated with *PTPL1* hypermethylation. This suggests that *PTPL1* is regulated by DNA methylation in lymphoma cells.

#### 5-Aza induces growth inhibition of Raji and Jurkat cells lines

**Figure 4:**
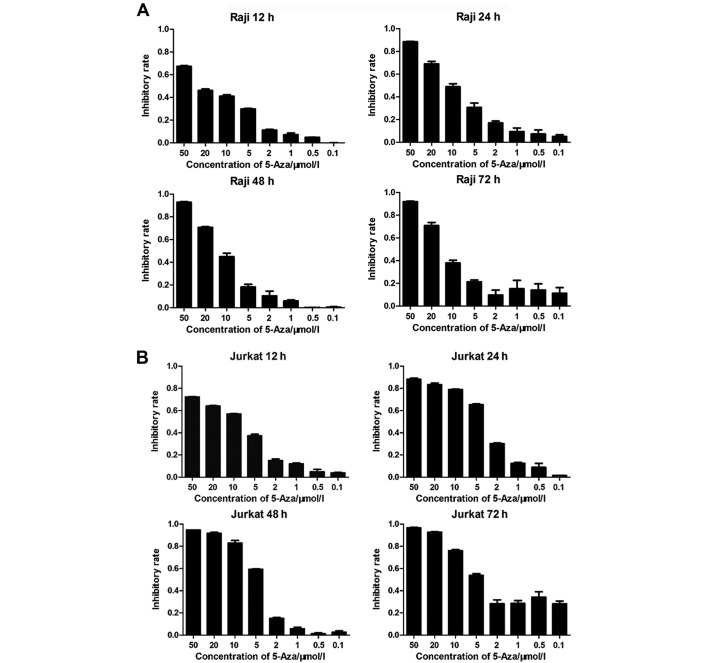
5-Azacytidine (5-Aza) induces growth inhibition of (A) Raji and (B) Jurkat cells lines.

Cell proliferation was detected using the CCK8 kit after 12, 24, 48 and 72 h treatment (Fig. 4). 5-Aza inhibited the proliferation of Raji and Jurkat cells in a concentration-dependent manner. Patterns of the inhibition efficiency differ in different cell lines.

#### Restoration of PTPL1 gene expression by 5-Aza, a DNMTs inhibitor

**Figure 5:**
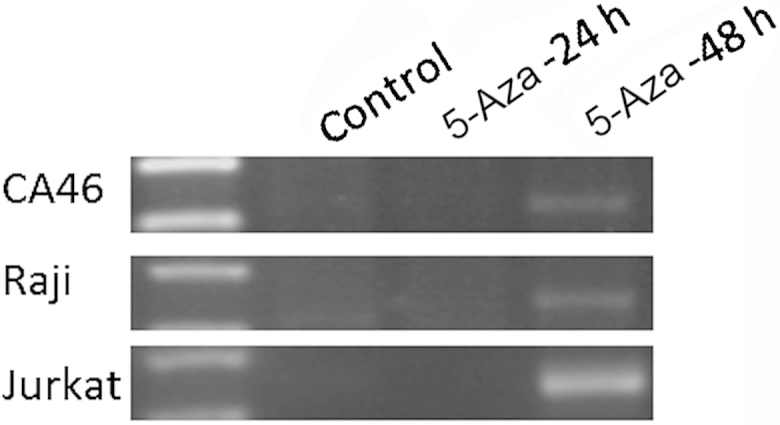
Restoration of *PTPL1* gene expression by 5-azacytidine (5-Aza). 5-Aza induced the re-expression of the *PTPL1*. Re-expression of *PTPL1* was observed after 48 h of treatment with 5-Aza.

*PTPL1* re-expression was investigated following treatment of CA46, Raji and Jurkat cells lines with the DNMTs inhibitor 5-Aza. 5-Aza treatment increased *PTPL1* mRNA expression compared to the untreated control in the cell lines. In CA46, Raji and Jurkat cells, treatment with 5-Aza lead to re-expression of *PTPL1* at 48 h (Fig. 5). The final half inhibitory concentrations were 20 *µ*M for CA46, 15 *µ*M for Raji and 3.5 *µ*M for Jurkat, respectively.

### Patients

#### Patient characteristics

Forty-seven samples were screened and 23 samples were followed up. Among the 23 follow-up patients, there were 11 males and 12 females, with a median age of 63 years (range, 26–81 years). Of the 23 patients, 5 (21.7%) were stage I, 6 (26.1%) were stage II, 2 (8.7%) were stage III, and 10 (43.5%) were stage IV. Using the Hans classification model, 9 cases were GCB and 14 were non-GCB, with a GCB:non-GCB ratio of 1:1.5 (Table I).

#### Promoter methylation status of DLBCL and reactive lymph node patients

**Figure 6:**
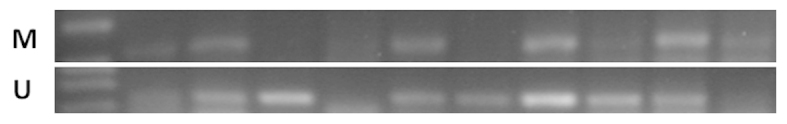
Methylation pattern of *PTPL1* in diffuse large B cell lymphoma patients.

Among the 47 DLBCL cases, the promoter of gene *PTPL1* was methylated in 59.6% (28/47) (Fig. 6), and unmethylated in 40.4% (19/47) (Table II).

In 9 GCB patients, the promoter of the *PTPL1* gene was methylated in 22.2% (2/9) and unmethylated in 77.8% (7/9). In the 14 non-GCB patients, the promoter of *PTPL1* was methylated in 64.3% (9/14) and unmethylated in 35.7% (5/14) (Table III). In the 16 reaction lymph node cases, the frequency of methylation was 6.3% (1/16), and the frequency of unmethylation was 93.8% (15/16).

#### Statistical analysis

The Fisher exact probability method was used to evaluate the difference of the number of methylated *PTPL1* promoters between DLBCL patients and reactive lymph node cases, GCB group and non-GCB group. The difference of the number of methylated *PTPL1* promoters between DLBCL patients and reactive lymph node proliferation cases was statistically significant (P<0.001). The difference of the number of methylated *PTPL1* promoters between the GCB and non-GCB group was not statistically significant (P=0.089).

## Discussion

The aim of the present study was to identify novel methylated biomarkers in lymphoma and to explore potential new therapeutic targets. The methylation pattern of the *PTPL1* gene was investigated in certain lymphoma-derived cell lines and 47 DLBCL cases. *PTPL1* was methylated in two Burkitt lymphoma cell lines (CA46 and Raji), one acute T cell lymphoma cell line (Jurkat) and one DLBCL cell line (DB); and unmethylated in the cutaneous T cell lymphoma cell line (Hut78), and in two mantle cell lymphoma cell lines (Maver and Z138). The methylated frequency of *PTPL1* in DLBCL patients was significantly higher compared to the non-malignant lymphoid control. Shi *et al* (13) reported that there were significant differences in DNA methylation between pre-germinal and germinal center-derived NHL. In general, germinal center-related lymphomas (follicular lymphoma and DLBCL) have more methylation compared to non-germinal center lymphoma (mantle cell lymphoma and chronic lymphocytic leukemia/lymphoma) (14). The present study shows that the *PTPL1* methylation frequency of non-GCB was higher compared with GCB. Clinically, the malignancy of non-GCB is higher compared with GCB, and prior to the appearance of rituximab, the prognosis of non-GCB was worse compared with GCB (15). Hypermethylation of the *PTPL1* promoter was also identified in a small number of carcinomas, including gastric and hepatocellular tumors, with 8/12 hepatocellular tumors presenting with significant methylation patterns (16). In addition, the methylation pattern of several genes were identified in lymphoma, such as SHP1, CD44, DAPK, GSTP1, MGMT, P14, P15, P16, P33, RB1, hMLH1, CDH1, APC, RASSFA1, TIMP3, VHL and BLU (17–20). Epigenetic abnormalities affecting histone-modifying enzymes and regulators, such as histone deacetylases (HDACs), have also been described in lymphoma (21). The methylation of lysine 9 and lysine 27 of histone H3 (H3K9me and H3K27me) can lead to transcriptional repression of the target gene; however, the methylation of lysine 4 and lysine 36 of histone H3 (H3K4me and H3K36me) can lead to transcriptional activation of the target gene (22,23). These all indicate that epigenetic alterations of gene expression are important in the development of tumorigenesis. The present study also confirmed this by showing that methylation of the promoter region of *PTPL1* correlates with lymphoma.

In addition, the present study has detected *PTPL1* mRNA in cell lines. To compare this finding with the methylation patterns of the previously described cell lines, the expression of *PTPL1* mRNA was ubiquitously expressed at different levels in the unmethylated cell lines (Hut78, Maver and Z138) and silenced in the total methylated cell lines (CA46, Raji, Jurkat and DB). Methylation of cytosine residues at CpG dinucleotides in the promoter region of genes is a major epigenetic modification in mammalian genomes and can lead to the silencing of gene expression (24,25). Epigenetic regulation of *PTPL1* expression was also documented in other cancers. In a study using a total of 82 tumor cell lines, Ying *et al* (26) showed that the expression of *PTPL1* was frequently downregulated or silenced in NHL (94%, 15/16), Hodgkin lymphoma (50%, 3/6), breast (30%, 3/10), gastric (60%, 6/10) and hepatocellular (67%, 8/12) carcinoma cell lines. In another study, Lee *et al* (27) identified that *PTPL1* can be detected in 80% of hepatocellular carcinoma with a significant variation of the protein expression level by immunohistochemistry staining. The present findings indicate that this epigenetic alteration of *PTPL1* is a common phenomenon in lymphoma and may be an important approach to inactivate cancer-related genes in this disease. However, these results also show that DNA methylation is not the only reason for *PTPL1* silencing.

The *PTPL1* re-expression pattern was also investigated following treatment with the DNMTs inhibitor 5-Aza to further confirm the role of DNA methylation in *PTPL1* regulation. Re-expression of *PTPL1* mRNA emerged at 48h after treated with 5-Aza. 5-Aza exerts its action by inhibiting DNA methylation (via its incorporation into DNA at cytosine positions) during DNA replication. In general, their transport is mediated by the human concentrative nucleoside transporter 1 (hCNT1) followed by their phosphorylation and conversion into their active tri-phosphate forms, namely 5-Aza-CTP (28). In this way, 5-Aza is able to interact with DNMTs, inhibit their activity and decrease overall DNA methylation levels. Therefore, the effect of 5-Aza on cell lines may be associated with the activity or expression of DNMT1, DNMT3A and DNMT3B (29). Overall, these data suggest that the DNA methyltransferase inhibitor 5-Aza was able to successfully lead to re-expression of *PTPL1* mRNA. The results confirmed that hypermethylation of *PTPL1* was responsible for gene silencing, as DNA demethylation resulted in reactivation of *PTPL1* transcription in the *PTPL1* hypermethylated cell lines. This may also support a tumor-suppressor role for *PTPL1* in lymphoma.

By contrast, the relative increase of *PTPL1* level in tumor tissues supports the role in tumor promotion. A high level of *PTPL1* mRNA expression in Kaposi's sarcoma, hepatocellular carcinomas, pancreatic adenocarcinomas, as well as with higher expression in T helper cells type 1 (which are resistant to apoptosis) versus T helper cells type 2 (which are sensitive to Fas ligand), also shows a correlation between tumor cell survival in the presence of *PTPL1* expression (11,30–32). In addition, investigators have shown relatively higher levels of *PTPL1* expression in multiple carcinomas compared to the normal adjacent tissue as detected by immunohistochemistry (33). Another previous study showed that in the process of dimethyl sulfoxide- and all-trans retinoic acid-induced differentiation in HL-60 cells, the increased resistance to death receptor-mediated apoptosis coincided with an increase in *PTPL1* (34). In CML, the resistance to death receptor-mediated apoptosis and the existence of leukemic stem cells were associated with an increase in *PTPL1* (35). A positive correlation between *PTPL1* expression and resistance to Fas-induced apoptosis has been shown in human T lymphotrophic virus (HTLV-I) infected T cell lines, ovarian cancer cell lines, human pancreatic cancer cell lines and squamous cell carcinomas of the head and neck cell lines (36). The presence of high *PTPL1* levels in tumor tissues may oppose *PTPL1* as a tumor suppressor. This may indicate that *PTPL1* has a role as a tumor promoter. The induction of *PTPL1* by an oncogene and relative increase of *PTPL1* levels in tumor tissues supports a role in tumor promotion. By contrast, epigenetic studies are more consistent with a role for *PTPL1* as a tumor suppressor. The impact of *PTPL1* on cancer is divided between its capacity to counteract the activity of oncogenic tyrosine kinases and its inhibitory interaction with the death receptor, Fas. The ability of *PTPL1* to inhibit signaling from growth factor receptors or oncogenes with tyrosine kinase activity can suppress tumor occurrence (37,38). By contrast, the ability of *PTPL1* to interact with the Fas receptor can promote tumor occurrence (39). Therefore, according to the tissue type and the cellular environment, different proportions of these two signaling pathways can lead to different biological effects. A complete understanding of epigenetic modifications of *PTPL1* and various *PTPL1* domains in mediating protein-lipid and protein-protein interactions will be critical in resolving the functional role of *PTPL1* in cancer. Establishing the precise function of *PTPL1* in NHL and understanding its mode of action will aid in our understanding of the use of *PTPL1* as a therapeutic target in NHL.

In the present study, the number of DLBCL cases was less, and that of subjects lost to follow-up was greater. More cases and future molecular studies are required to determine the role of *PTPL1* methylation in the development and progression of NHL.

In conclusion, the study showed that *PTPL1* expression is regulated by DNA methylation, not only in lymphoma cell lines, but also in the DLBCL patients. The loss of *PTPL1* mRNA is the consequence of *PTPL1* methylation and can be reversed by 5-Aza. Thus, 5-Aza may be further investigated as a novel therapeutic agent for NHL.

## Figures and Tables

**Table I:** Clinical characteristics of 23 patients with DLBCL.

Clinical characteristic	Patients, n (%)
Gender	
Male	11 (47.8)
Female	12 (52.2)
Age, years	
<65	15 (65.2)
≥65	8 (34.8)
Stage	
I	5 (21.7)
II	6 (26.1)
III	2 (8.7)
IV	10 (43.5)
Type	
GCB	9 (39.1)
Non-GCB	14 (61.9)

DLBCL, diffuse large B cell lymphoma; GCB, germinal center phenotype.

**Table II:** *PTPL1* methylation pattern in DLBCL patients.

Patients	Methylated, n (%)	Unmethylated, n (%)
DLBCL, n=47	28 (59.6)	19 (40.4)
Reactive lymphnodes, n=16	1 (6.3)	15 (93.7)

Fisher's exact test P<0.001. DLBCL, diffuse large B cell lymphoma.

**Table III:** *PTPL1* methylation pattern in GCB and non-GCB patients.

Patients	Methylated, n (%)	Unmethylated, n (%)
GCB, n=9	2 (22.2)	7 (77.8)
Non-GCB, n=14	9 (64.3)	5 (35.7)

Fisher's exact test P=0.089. DLBCL, diffuse large B cell lymphoma; GCB, germinal center phenotype.
